# Early-life Stress, Depressive Symptoms, and Inflammation: The Role of Social Factors

**DOI:** 10.1093/geroni/igaa057.3380

**Published:** 2020-12-16

**Authors:** Julia Nakamura, Eric Kim, Kelly Rentscher, Kate Kuhlman

**Affiliations:** 1 University of British Columbia, Vancouver, British Columbia, Canada; 2 University of California, Los Angeles, Los Angeles, California, United States; 3 University of California, Irvine, Irvine, California, United States

## Abstract

Early-life stress (ELS) is associated with elevated risk of adverse psychological (e.g., depression) and physical health outcomes (chronic diseases driven by inflammation) in older adulthood. We evaluated whether four social factors buffered the ELS-depressive symptoms and ELS-inflammation associations. Data were from 3,416 adults (58.28% female; Mage=68.41; SDage=10.24) who participated in the 2006 wave of the Health and Retirement Study, a nationally representative sample of older adults in the United States. We used hierarchical regression analyses to first test the main effects of ELS on depressive symptoms and inflammation (high-sensitivity C-reactive protein). We then assessed whether four social factors (perceived support, frequency of social contact, network size, and volunteer activity) moderated the ELS-depressive symptoms and ELS-inflammation relationships. We found a small positive association between ELS and depressive symptoms (B=0.17, SE=0.05, p=.002), which was moderated by social contact and perceived support. Specifically, ELS was only associated with elevated depressive symptoms for participants with limited social contact (B=0.24, SE=0.07, p<.001) and low perceived support (B=0.24, SE=0.07, p<.001). These associations remained after accounting for potential confounders (age, body-mass index, adulthood stress, and marital status). ELS was not associated with inflammation, and no social factors moderated the ELS-inflammation link. Increased social contact and perceived support may be protective for individuals at an elevated risk of developing depressive symptoms as a result of ELS. Future interventions may benefit from leveraging these social factors to improve quality of life in adults with ELS.

